# Sex and gender considerations in reporting guidelines for health research: a systematic review

**DOI:** 10.1186/s13293-021-00404-0

**Published:** 2021-11-20

**Authors:** Amédé Gogovor, Hervé Tchala Vignon Zomahoun, Giraud Ekanmian, Évèhouénou Lionel Adisso, Alèxe Deom Tardif, Lobna Khadhraoui, Nathalie Rheault, David Moher, France Légaré

**Affiliations:** 1 Quebec SPOR-SUPPORT Unit, Quebec City, QC Canada; 2 Tier 1 Canada Research Chair in Shared Decision Making and Knowledge Translation, Université Laval, Quebec City, QC Canada; 3 Department of Family Medicine and Emergency Medicine, Université Laval, Quebec City, QC Canada; 4 VITAM-Centre de Recherche en Santé Durable, Pavillon Landry-Poulin, 2525, Chemin de la Canardière, Quebec, QC G1J 0A4 Canada; 5 School of Epidemiology and Public Health, University of Ottawa, Ottawa, ON Canada; 6 Centre for Journalology, Clinical Epidemiology Program, Ottawa Hospital Research Institute, The Ottawa Hospital, General Campus, Centre for Practice Changing Research Building, 501 Smyth Road, PO Box 201B, Ottawa, ON K1H 8L6 Canada

**Keywords:** Reporting guideline, Sex, Gender, Quality of reporting, Systematic review, Health research

## Abstract

**Background:**

Despite growing recognition of the importance of sex and gender considerations in health research, they are rarely integrated into research design and reporting. We sought to assess the integration of sex, as a biological attribute, and gender, as a socially constructed identity, in published reporting guidelines.

**Methods:**

We conducted a systematic review of published reporting guidelines listed on the EQUATOR website (www.equator-nework.org) from inception until December 2018. We selected all reporting guidelines (original and extensions) listed in the EQUATOR library. We used EndNote Citation Software to build a database of the statements of each guideline identified as a "full bibliographic reference" and retrieved the full texts. Reviewers independently extracted the data on use of sex and gender terms from the checklist/abstract/main text of guidelines. Data were analyzed using descriptive statistics and narrative synthesis.

**Results:**

A total of 407 reporting guidelines were included; they were published between 1995 and 2018. Of the 407 guidelines, 235 (57.7%) mentioned at least one of the sex- and gender-related words. In the checklist of the reporting guidelines (*n* = 363), “sex” and “gender” were mentioned in 50 (13.8%) and 40 (11%), respectively. Only one reporting guideline met our criteria (nonbinary, appropriate categorization, and non-interchangeability) for correct use of sex and gender concepts. Trends in the use of "sex" and "gender" in the checklists showed that the use of “sex” only started in 2003, while “gender” has been in use since 1996.

**Conclusions:**

We assessed the integration of sex and gender in reporting guidelines based on the use of sex- and gender-related words. Our findings showed a low use and integration of sex and gender concepts and their incorrect use. Authors of reporting guidelines should reduce this gap for a better use of research knowledge.

*Trial registration* PROSPERO no. CRD42019136491.

**Supplementary Information:**

The online version contains supplementary material available at 10.1186/s13293-021-00404-0.

## Highlights

- Omission of sex and gender considerations is a recurring deficiency in research design and reporting
- Integration of sex and gender considerations in health research reporting guidelines is very low
- Three criteria were used to assess correct use of sex and gender concepts
- Only one reporting guideline met the three criteria
- A call to action is made to address these deficiencies

**Supplementary Information:**

The online version contains supplementary material available at 10.1186/s13293-021-00404-0.

## Introduction

Deficiencies in the quality of reporting of health research are well documented in the literature [1, 2]. Consequences of inadequate reporting include lapses of scientific integrity and difficulty in judging the reliability of the results and the relevance of the evidence [2].

One of the recurrent deficiencies in research design and reporting is the lack of the integration of sex and gender considerations. Despite growing recognition of the importance of sex and gender in the manifestation and management of health conditions, their consideration is rarely integrated in research design and reporting [3–5]. This limitation may further explain why there is waste in research, as research being performed right now is not aligned with or does not reflect the sex and gender profiles of the population. Solutions include mandating requirements and policies to integrate sex and/or gender in health research by funding agencies and publishers [6–9].

Based on the knowledge-to-action process [10], synthesizing gaps in the integration of sex and gender and developing appropriate reporting guidelines will contribute to effective knowledge translation. Sex refers to “a set of biological attributes in humans and animals and is primarily associated with physical and physiological features, including chromosomes, gene expression, hormone levels and function, and reproductive/sexual anatomy” [11]. The traditional categorization of sex is dichotomous as male or female; sometimes, other response options are offered (intersex, other). Gender refers to “the socially constructed roles, behaviours, expressions and identities of girls, women, boys, men, and gender diverse people. "Gender" influences how people perceive themselves and each other, how they act and interact, and the distribution of power and resources in society” [11]. Categories of gender include men, women, and gender-diverse people. The term gender-diverse adopted in North America includes a broad range of identities (e.g., transgender and two-spirit) and might have different cultural meanings globally [12]. Thus, “sex” and “gender” hold different meanings and should not be used interchangeably [13–15].

Reporting guidelines are developed to improve the transparency, accuracy and completeness of reporting for different types of research [16, 17]. Thus, where relevant, these guidelines need to incorporate items related to sex and gender to provide comprehensive guidance. A reporting guideline is defined as “a checklist, flow diagram, or explicit text to guide authors in reporting a specific type of research, developed using explicit methodology” [2]. A recent study examined the inclusion of sex and gender considerations in the publishing guidelines of several top-ranking health journals, and the study made recommendations to strengthen these considerations in the policies and practices of health journals [18]. However, no systematic investigation of sex and gender considerations in reporting guidelines has been made. The aim of this study was to assess the integration of sex and gender concepts in published health research reporting guidelines based on the use of sex- and gender-related words. We examined the correct use of sex and gender terms, the publication trends in the use of sex and gender terms, and the nature of sex and gender information in the checklist.

## Methods

The protocol of the systematic review is registered with the International Prospective Register of Systematic Reviews (PROSPERO CRD42019136491) [19]. We followed the Preferred Reporting Items for Systematic Reviews and Meta-Analyses (PRISMA) statement [20] to guide the report (Additional file 1).

### Eligibility criteria, information sources and study selection

The EQUATOR Network Team, which maintains a collection of reporting guidelines for health research, has developed search strategies for PubMed, Embase, CINAHL, and Web of Science to identify reporting guidelines published since 1996 in English. Free-text and controlled vocabulary terms used in the search strategies included reporting guideline(s); reporting standard(s); reporting guidance; reporting requirement(s); reporting criteria; reporting recommendation(s); reporting checklist(s); reporting statement(s) and reporting instruction(s). The search strategies are run regularly [21], and the results are systematically reviewed by the EQUATOR Network Team for inclusion in the database of reporting guidelines.

We systematically included all published and listed reporting guidelines (original and extensions) in the Equator Network registry (www.equator-nework.org), as of 31 December 2018; thus, a selection flowchart was not applicable. We used EndNote (EndNote Citation Software, Version 9.3, Clarivate Analytics, New York, NY, USA) to build a database of the statement of each reporting guideline identified as a “full bibliographic reference” on EQUATOR and retrieved the full texts. This allowed us to be consistent by having one main document (statement) per reporting guideline and was justified by the fact that users rarely consult explanation and elaboration documents [22]. For reported guidelines that included sex and gender terms in their checklists, we consulted complementary documents listed on their webpage in the EQUATOR Network Registry to complete the assessment of correct use of these terms.

### Data collection process

Pairs of reviewers (AG, GE, ÉLA, ADT) independently extracted study data using a pretested extraction form. The information extracted included the following: (1) Characteristics of the reporting guidelines (e.g., author, year, title, acronym, type of study as documented on EQUATOR [randomized trial, observational, systematic review, protocol, diagnostic/prognostic, case report, clinical practice guideline, qualitative research, animal preclinical, quality improvement, economic evaluation, experimental, other, and ‘nonspecific’ for reporting guidelines that do not apply to any specific type of study]); (2) Use of sex and gender terms, i.e. presence of any sex- or gender-related words [sex, gender, female(s), male(s), man, woman, men, women, boy(s), girl(s), gender-diverse]) in the checklist, flowchart and main text and the number of occurrences of these words in each (using electronic text search tools). Nonbinary words (e.g., intersex and gender-diverse) were captured by searching “sex” and “gender.” For scanned documents and images, we used a free online Optical Character Recognition (OCR) tool to convert them into editable text. The main text (statement) included text from introduction to conclusion. We also extracted sex- and gender-related words from the abstract and the reference list of reporting guidelines included. Sex- and gender-related words in the following sections were excluded: affiliation, acknowledgment, tables, and figures that were not flowcharts. (3) Definitions of "sex" and "gender" were based on established definitions [11, 23]. The correct use of sex and gender was assessed using three criteria: nonbinary use (more than two categories), use of appropriate categories (e.g., male/female/intersex for sex and man/woman/gender-diverse for gender), and noninterchangeable use of sex and gender. The three criteria are described elsewhere [24] and summarized in Table 1. The use of sex and gender terms was considered correct if all three criteria were met, incorrect if at least one of three criteria was not met, and unclear if at least one of the criteria was reported as unclear and the others were met. We consulted explanation and elaboration documents of reported guidelines for the assessment of correct use of sex and gender concepts in their checklists when available. (4) Type of sex and gender information in the checklist. In addition, a sample of 10% of the reporting guidelines was searched manually and reported (Additional file 2: Fig. S1) for comparison with the electronic search in the following sections: checklist, abstract, statement, and references. We contacted the author of one reporting guideline and obtained a copy of the checklist because a supplemental document was not available. Discrepancies were resolved by consensus between two team members, and with a third member when necessary. We performed double counting to resolve discrepancies between electronic versus manual searches.

**Table 1:** Criteria for assessing correct use of sex and gender terms in reporting guidelines

Criterion	Definition
Nonbinary	1. Nonbinary use: male, female or intersex for sex; man (men), woman (women) or gender-diverse for gender; description of sex or gender implies more than two categories 2. Binary-use: male or female for sex; boy/man/men or girl/woman/women for gender; description of sex or gender implies two categories 3. Unclear: terms of sex or gender used without specification of categories
Appropriate categories	1. Appropriate: consistent use of male/female/intersex for sex; boy/man/men, girl/woman/women or gender-diverse for gender 2. Inappropriate: inconsistent use of male/female/intersex for sex; boy/man/men, girl/woman/women or gender-diverse for gender; e.g., male/female/intersex for gender 3. Unclear: terms of sex or gender used without specification of categories
Non-interchangeability	1. Noninterchangeable: consistent use of sex to describe biological attributes and gender for sociocultural attributes 2. Interchangeable: inconsistent use of sex to describe biological attributes and gender for sociocultural attributes; e.g., indiscriminate use of sex and gender, male and man for the same concept 3. Unclear: terms of sex or gender used without specification of categories, any other situation, where assessment is unrealizable

Adapted from Adisso et al. [24]

### Internal validity assessment

The assessment of internal validity ensures the minimization of potential bias in the development of reporting guideline recommendations. To assess the quality of the included reporting guidelines, we adapted the risk of bias checklist developed by Cukier et al. [25], since there is no validated tool for internal validity assessment for methods systematic reviews. One author (AG) first drafted a list of the three criteria relevant to reporting guideline development [2]. The list of items was reviewed by an author (DM) with extensive experience in the development of reporting and pilot tested (two rounds) by members of the author group for consistency and feasibility. The final 3-item internal validity assessment checklist is shown in Box 1 (detailed coding manual in Additional file 3). Each item was coded “yes,” “no,” or “unclear.” The judgment rule to determine evidence-based development of the included reporting guidelines was high internal validity (i.e., low risk of bias) if  ≥ 2 “yes.” The assessment was based on the main text (statement) of each reporting guideline. For criterion 1, when a specific group was named the developer of the guideline, we consulted the internet to determine whether it represented more than one stakeholder group. Pairs of three reviewers (AG, GE, HTVZ) independently conducted the assessment and any disagreements were resolved by consensus or third-party adjudication.

Box 1. List of quality assessment criterion

1. Did the developers of the guideline represent more than one stakeholder group (e.g., researchers, funders, publishers)?
2. Did the developers report gathering any data for the creation of the guideline (e.g., carry out a literature review, collect anecdotal data)?
3. Did the developers report the use of a consensus process (e.g., Delphi, RAND/UCLA Appropriateness Method, nominal group technique, consensus meeting, development conference)?

### Data synthesis and analysis

We analyzed extracted data using descriptive statistics and reported the numbers and percentages of reporting guidelines that integrated sex- and/or gender-related items. We also calculated the mean number of occurrences of sex- or gender-related words in the different sections of reported guidelines, and we calculated the mean number of sex and gender terms in the checklists for each year of publication. We reported publication trends over time in the use of sex and gender terms in the checklists. For the reporting guidelines that integrated sex and/or gender terms in their checklists, we calculated the percentage of reporting guidelines that met each of the three criteria for correct use; and the percentage with correct use of sex and gender terms, i.e., all three criteria were met.

We qualitatively synthesized the type of sex and gender information in the checklist into three groups: (i) mention of sex and/or gender terms with no description of the categories; (ii) use of sex or gender terms with description of the categories; and (iii) detailed definition or description of how sex and/or gender should be integrated. Finally, we compared the frequencies of the use of sex- and gender-related words for electronic and manual modes of assessment. We identified the number of times the terms of interest were used throughout the different sections of reporting guidelines. This identification was done both electronically and manually to ensure consistency of our results. There was concordance if the numbers were the same. SAS (SAS, version 9.4, Institute, Cary, NC, USA) and R (R software, version 3.6, R Core Team, University of Auckland, New Zealand) were used to perform the analyses.

## Results

### Characteristics of the included reporting guidelines

**Fig. 1:**
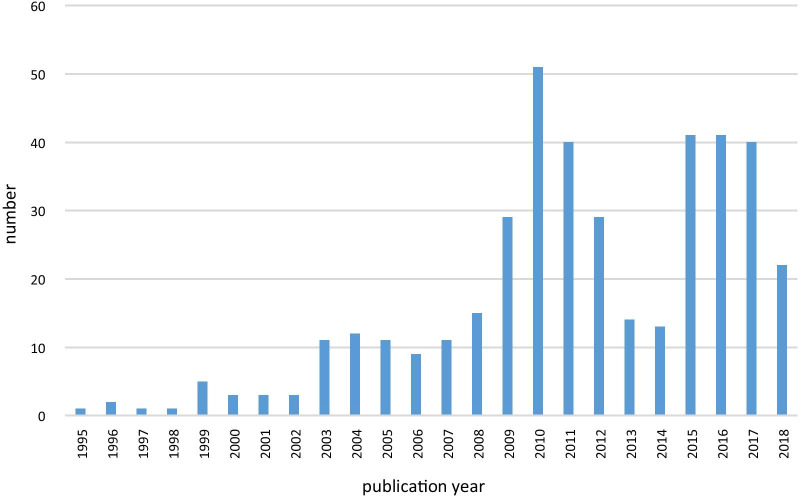
Distribution of reporting guidelines per year

A total of 407 reporting guidelines (statements) were included in the review, published between 1995 and 2018. Seven related explanation and elaboration documents were consulted during the assessment of correct use of “sex” and “gender.” The most prevalent year of publication was 2010 with 51 reported guidelines (Fig. 1). Of the 407 reporting guidelines included, 66 (16.2%) were extensions of existing guidelines, 363 (89.2%) included a checklist and 26 (6.4%) a flowchart. While 349 (85.8%) included an abstract, only 134 (33%) out of 407 reporting guideline statements adopted the traditional structure of a scientific article with the following sections: introduction, methods, results, and conclusion. Most reporting guidelines 243 (59.7%) were developed for specific methodological approaches (for part of/whole report), while 122 (29.9%) were developed for a specific type of study. Based on the classification on the EQUATOR website, the most common study types targeted by the included reported guidelines were "experiment" (145, 35.6%), "randomized trials" (132, 32.4%), and "observational" (115, 28.3%) (Table 2). A reference list of included reporting guidelines is shown in Additional file 4: Table S1.

**Table 2:** Distribution of reporting guidelines by study type

Study type	*N* (407)^a^	%
Case report^b^	4	1.0
Clinical practice guideline	8	2.0
Diagnostic/prognostic	18	4.4
Economic evaluation	16	3.9
Experiment	145	35.6
Nonspecific^c^	103	25.3
Observational	115	28.3
Other^d^	16	3.9
Preclinical	15	3.7
Protocol	10	2.5
Quality improvement	5	1.2
Qualitative	17	4.2
Randomized trial	132	32.4
Systematic review	34	8.4

^a^Numbers do not add up to *N*, because reporting guidelines can be classified in more than one category

^b^Based on the category of study types on EQUATOR homepage

^c^Do not apply to any specific type of study

^d^As specified on the individual page of reporting guidelines on EQUATOR

### Electronic versus manual search of words

A total of 41 (10%) reporting guidelines were randomly selected for the comparison of electronic and manual identification of sex- and gender-related words. We found 17 discrepancies in 12 reporting guidelines and only one discrepancy was in favor of manual search, after verification (Additional file 5: Table S2). The concordance between the electronic and manual searches for "sex," "gender" and related words was 97.6% for “sex,” “women” and “men” and 100% for “gender,” “female,” “male,” “woman,” “man,” “boy,” and “girl” in the checklists. In the statements, the concordance was 95.1% for “gender,” “women,” and “men”; 97.6% for “male”; and 100% for “sex,” “man,” “female,” “boy” and “girl.” The references section showed a concordance of 95.1% for “sex”; 97.6% for “gender,” “women,” and “men”; and 100% for “woman,” “man,” “female,” “male,” “boy” and “girl”. No discrepancies were found in the abstract section (Additional file 6: Table S3).

### Internal validity assessment

**Fig. 2:**
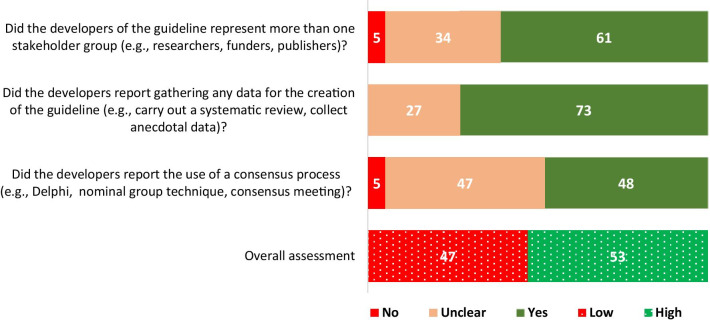
Summary of internal validity assessment

We conducted the internal validity assessment on a random number of 100 reported guidelines of 407 included due to time and resource constraints. The summary of the assessment is presented in Fig. 2 (Additional file 7). There was evidence-based development of just above half the assessed reporting guidelines, i.e., high internal validity (53/100).

### Presence of sex- and gender-related words

Of 407 reporting guidelines, 235 (57.7%) mentioned at least one of the sex- and gender-related words (sex, gender, female(s), male(s), man, woman, men, women, boy(s), girl(s), gender-diverse). The distribution of the use of sex- and gender-related words in the different sections of reporting guideline statements is shown in Table 3. In the checklist of the reporting guidelines (*n* = 363), only 50 (13.8%) used “sex,” 40 (11%) used “gender,” 86 (23.7%) used “sex” and/or "gender," and 4 (1.1%) used both (Additional file 8: Figure S1). In the main text (statement), “sex” and “gender” were mentioned in 14.2% and 17.9%, respectively. The most common sex- and gender-related words used in the main text (statement) were “gender” (73/407, 17.9%), “woman/women” (64/407, 15.7%), “sex” (58/407, 14.2%), and “male(s)” (42/407, 10.3%). Distributions of the use of sex and gender terms according to study types and sections of reported guidelines are presented in Additional file 9: Table S4 and Additional file 10 Table S5).

**Table 3:** Percentage of the presence of sex- and gender-related words in different sections of reporting guidelines

	Checklist *N* = 363	Flowchart *N* = 26	Abstract *N* = 349	Statement *N* = 407	References *N* = 407	Overall *N* = 407
Sex	50 (13.8)	1 (3.9)	3 (0.9)	58 (14.2)	27 (6.6)	103 (25.3)
Gender	40 (11)	1 (3.9)	5 (1.4)	73 (17.9)	19 (4.7)	96 (23.6)
Female(s)	10 (2.8)	1 (3.9)	2 (0.6)	39 (9.6)	13 (3.2)	51 (12.5)
Male(s)	9 (2.5)	1 (3.9)	2 (0.6)	42 (10.3)	15 (3.7)	55 (13.5)
Man/men	7 (1.9)	0 (0.0)	5 (1.4)	39 (9.6)	39 (9.4)	18 (4.4)
Woman/Women	1 (0.3)	0 (0.0)	1 (0.3)	11 (2.7)	2 (0.5)	54 (13.3)
Women	6 (1.7)	1 (3.9)	4 (1.1)	64 (15.7)	65 (15.9)	93 (22.9)
Boy(s)	0 (0.0)	0 (0.0)	0 (0.0)	1 (0.1)	4 (1.0)	5 (1.2)
Girl(s)	0 (0.0)	0 (0.0)	0 (0.0)	6 (1.5)	5 (1.2)	9 (2.2)
Gender diverse	0 (0.0)	0 (0.0)	0 (0.0)	1 (0.1)	0 (0.0)	1 (0.2)
No sex- or gender-related words	270 (74.3)	23 (88.5)	337 (96.6)	241 (59.2)	294 (72.2)	172 (42.3)

Distributions are reported as *n* (%). "Statement" refers to the main text from introduction to discussion/conclusion, excluding tables, figures, acknowledgement, and affiliations. No “intersex” was found so it is not included in the table. Percentages do not equal 100% because sections can include more than one word

### Correct use of sex and gender terms

Table 4 reports the correct use of sex and gender terms in the reporting guidelines that used these terms in their checklist (*n* = 86). The use was correct in only one reporting guideline published in 2016 (all three criteria met) (Additional file 4: Table S1 RG156), incorrect in 23 (26.7%) and unclear in 62 (72.1%). Few reporting guidelines met the individual criteria: 4 (4.7%) met criteria for nonbinary use: use of “gender-diverse” in one guideline (Additional file 4: Table S1, RG156), “transgender” in two (Additional file 4: Table S1, RG156, RG196), and other categories in two (Additional file 4: Table S1, RG33, RG92); 5 (5.8%) met the noninterchangeable criteria (Additional file 4: Table S1, RG134, RG156, RG194, RG329, RG386); and 9 (10.5%) met criteria for the use of appropriate categories (Additional file 4: Table S1, RG134, RG140, RG141, RG156, RG194, RG225, RG373, RG386, RG406).

**Table 4:** Correct use of sex or gender terms in reporting guidelines that included these terms in their checklist

Criteria	*N* (86)	Percentage, %
Nonbinary	4	4.7
Binary	16	18.6
Unclear	66	76.7
Appropriate	9	10.5
Inappropriate	15	17.4
Unclear	62	72.1
Noninterchangeable	5	5.8
Interchangeable	18	20.9
Unclear	63	73.3
Correct	1	1.2
Incorrect	23	26.7
Unclear	62	72.1

### Trends in the use of sex and gender terms

**Fig. 3:**
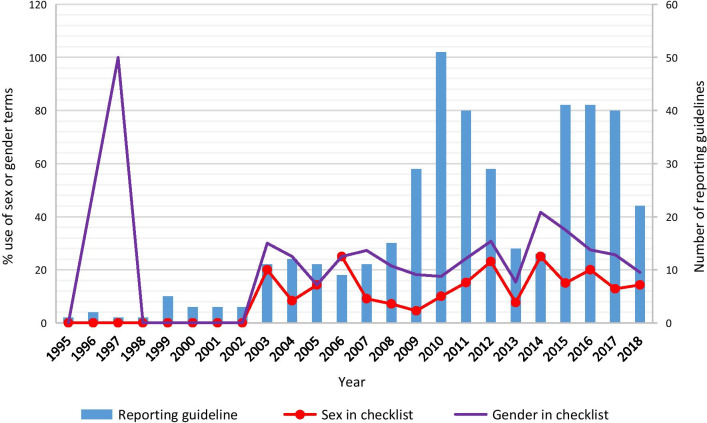
Publication trends in the use of sex and gender terms in checklist

Publication trends (from 1995 to 2018) in the use of sex and gender terms in checklists showed that “sex” and “gender” were increasingly used over time (Fig. 3) based on the proportion of reporting guidelines. A similar trend was observed when considering the mean number of “sex” and “gender” occurrences in the checklists (Additional file 11: Fig. S2). While “gender” has been used since 1996, the use of “sex” started only in 2003.

### Type of sex and gender information in the checklist

Of the reporting guidelines that mentioned “sex” and/or “gender” in their checklist, only two provided detailed information about “sex” (Additional file 4: Table S1, RG156 and RG303) or “gender” (Additional file 4: Table S1, RG156). Two and four reporting guidelines specified the categories for “sex” and “gender” respectively. The remaining vast majority mentioned sex or gender terms only in the list of demographic or baseline information required.

## Discussion

With the growing recognition of the importance of sex and gender considerations in research design and reporting, it is important to identify gaps in the integration of sex or gender considerations in reporting guidelines. We assessed the integration of sex and gender considerations in published reporting guidelines based on the use of sex and gender related words. At least one sex- and gender-related word was mentioned in 57.3% of the reporting guidelines assessed. In the checklist, which we have labelled as the most important section of the reporting guidelines, only 14% and 11% used “sex” or “gender” respectively, while 14% used “sex” and 18% used “gender” in the main text (statement). Only one reporting guideline correctly used “sex” and “gender” based on the criteria, and inclusion of nonbinary categories was the least met criterion. Overall, sex and gender terms were increasingly used over time with an earlier use of “gender” than “sex.” Finally, only two reporting guidelines provided detailed information about sex or gender terms in their checklist. These findings lead us to the following observations.

We found that only a small number of reporting guidelines used sex and gender terms, particularly in their checklists. Studies that examined the use of sex- and gender-related words in published health research articles reported similar findings. In a 2019 review that described considerations of sex and gender in 113 Cochrane reviews of interventions for preventing healthcare-associated infections (published between 2003 and 2016), 51% and 37% used “sex” and “gender” respectively [26]. In another study that examined original investigations on diabetes published in 2015 in the ten highest-impact general medicine and diabetes-specific journals, “sex” and “gender” were mentioned in the introduction sections of 10% and in the methods sections of 30% [27]. More recently, “sex” was mentioned in 43% and “gender” in 41% of 87 studies (published between 1995 and 2017) included in a Cochrane review on the effectiveness of interventions for increasing the use of shared decision making by health professionals [24]. Thus, the proportion of reporting guidelines (published 1995 to 2018) that use of sex and gender terms, according to our findings, is far below that of original studies (14% versus 43% for “sex”; 18% versus 41% for “gender”) published during the same time period. Reporting guidelines are published as scientific articles that recommend the minimum elements required to adequately report different types of studies. Researchers, funders, and journal editors are among the end-users of reporting guidelines [2, 28], and there have been significant advances in recognizing the importance of sex and gender. For example, awareness-raising articles and reports [29–33], requirements for grant applicants from top funders (Canadian Institutes of Health Research, the European Commission, and the US National Institutes of Health) [6–8], and specific instructions regarding sex and gender considerations by journal editors [34, 35]. Given the dates of these requirements (around 2016), it is worth noting that the only reporting guideline that met all three criteria of correct use was published in 2016 [29]. Thus, while the use of sex and gender terms has slightly increased (Fig. 3), it may be too early to notice any impact on their correct use. Authors of reporting guidelines should play their part by integrating items regarding appropriate sex and gender considerations.

Only one reporting guideline met all three criteria of the correct use of sex and gender concepts [29]. This is consistent with previous findings of studies that examined sex and gender considerations in health research, regardless of how “correct” or “appropriate” use was defined. In a previous review study, from which we adopted the definition of correct use (based on three criteria), no study met all three criteria [24]. Tannenbaum et al. found that only 35% of Canadian clinical practice guidelines (published between 2013 and 2015 for noncommunicable health conditions) that included “sex” and/or “gender” used the terms correctly [5] according to the Sex and Gender Equity in Research guidelines [29]. This result was expected, because only recently (in the 2010s) has a clear distinction between sex and gender, particularly in health research, reached the mainstream [7, 29, 31, 32, 36–42]. Indeed, we found a modest increase in the use of “sex” and “gender” over time. The earlier use of “gender” in reporting guidelines is not surprising and may have been used to mean “sex”, illustrating the longstanding inadequate and interchangeable use of these terms, particularly use of “gender,” as reported elsewhere [5, 24, 26, 27, 43]. The correct terms should be used in the future development of reporting guidelines or updates of existing guidelines. Authors can cross-reference SAGER guidelines or integrate them by including examples of good practice for items relevant to their reporting guidelines [29].

Another gap relates to the nature of the sex and gender information provided. In our study, detailed information was provided in only two checklists. As in similar studies [24, 26, 27], we were unable to assess the correct use of “sex” and “gender” for the vast majority of checklists (reporting guidelines) because of insufficient or inadequate information. This occurred even after we consulted the explanation and elaboration documents of the reporting guidelines that used “sex” and “gender” terms in their checklists (Table 4). Reporting guidelines are published as an article (statement) that most often includes a clear checklist of items to report. Following the publication of the Guidance for Developers of Health Research Reporting Guidelines (recommended steps for the development of reporting guidelines) [2], an increasing number of authors of reporting guidelines have published companion explanation and elaboration documents. However, it is documented that very few users actually consult those companion documents, hence we suggest including more tailored and expanded details in the checklists themselves of published reporting guidelines [22]. The proposed "change of paradigm" would help authors of reporting guidelines to seize this opportunity to improve the integration of sex and gender by providing specific instructions on how to consider sex and/or gender within the different sections at the stage of writing manuscripts. Alternatively, authors of reporting guidelines could harness the potential of information technology and work with the EQUATOR Network to integrate their reporting checklists into the platform that its partner company, Penelope, is developing to help authors identify relevant reporting guidelines. Indeed, the platform offers the flexibility to insert hypertexts and hyperlinks to detailed information [44].

Our study has some limitations. First, our systematic review was based on reporting guidelines in the Equator Network registry; thus, we cannot guarantee that all published reporting guidelines of health research were included. However, we are confident that all relevant reporting guidelines were included given the rigorous processes of selection and curation by the EQUATOR Network Team. Second, we used the main document of reporting guidelines (statement) to assess the use of sex- and gender-related words and consulted only available explanation and elaboration documents for the reporting guidelines that used “sex” and “gender” terms in their checklists to assess correct use of these terms. This choice was justified by the fact that the checklist is the most important section of reporting guidelines, and users rarely consult explanation and elaboration documents [22]. The comparison of electronic and manual counts of sex- and gender-related words showed that the electronic count, while not perfect, was the most accurate and is very unlikely to affect our assessment. We assessed evidence-based development of a sample of reporting guidelines due to practical constraints. However, using a random sample, we are confident that our results reflect the current quality of published reporting guidelines, with almost half of them not developed according to the recommended steps [2]. It is unclear whether this finding impacts the use of sex- and gender-related words in reporting guidelines. Finally, we assessed the presence of sex- and gender-related words but not how considerations of sex and gender were integrated into the guidelines. The appropriate level of sex and gender information in reporting guidelines remains to be determined. While the focus of this study is sex and gender considerations, it is worth mentioning the progressing consensus to examine multiple intersecting identities that impact health outcomes [18, 43]. We can make the following recommendations:

- End users of current reporting guidelines should check the use of sex and gender terms against the standard definitions and make the necessary corrections for their appropriate use when writing their manuscripts; they should also refer to SAGER guidelines [29].
- Journal editors should provide guidelines for transparent reporting of sex and gender [35]. This has been implemented by some journals, such as JAMA [9].
- Authors of current reporting guidelines with obvious misuse of sex and gender concepts and related terms should address it by updating their statement (Additional file 4: Table S1).
- The EQUATOR Network should encourage developers of reporting guidelines to consider sex and gender by updating their guidance [45] and provide them with more operative information, including the use of SAGER [29].

### Perspectives and significance

An important aspect of the awareness of sex and gender considerations in all aspects of health research is the issue of incorrect use of these concepts. This review of sex and gender in reporting guidelines for health research informs their developers of the first action needed to improve the reporting practices for sex and gender information. Improvement could be assessed through a trend analysis of successive versions of reporting guidelines with respect to the integration and correct use of sex and gender concepts. Future research should define criteria to assess the relevance and the appropriate level of sex and gender information in reporting guidelines for health research.

## Conclusion

The use of sex- and gender-related words is rare in published reporting guidelines, particularly in their checklists. The initial step for authors of reporting guidelines remains to address the issues of inappropriate use of the terms “sex” and “gender.” Our findings will inform developers and users of these guidelines and may ultimately help reduce this gap for a better use of research knowledge and quality of evidence synthesis from health research.

## Supplementary Information

**Additional file 1.** PRISMA checklist.

**Additional file 2: Fig. S1.** Example of manual count of sex- and gender-related words.

**Additional file 3.** Validity assessment for methods systematic review.

**Additional file 4: Table S1.** List of included reporting guidelines.

**Additional file 5. Table S2.** Comparison of electronic and manual identification of sex and gender related words in reporting guidelines.

**Additional file 6. Table S3.** Concordance between electronic and manual identification of sex and gender related words in reporting guidelines.

**Additional file 7.** Detailed internal validity assessment.

**Additional file 8: Fig. S1.** Use of sex and/or gender terms in the checklists.

**Additional file 9: Table S4.** Distribution of the use of “sex” in various study types and sections of reporting guidelines.

**Additional file 10: Table S5.** Distribution of the use of “gender” in various study types and sections of reporting guidelines.

**Additional file 11: Fig. S2.** Publication trends in the mean number of occurrences of "sex" and "gender" in the checklist.

**Additional file 12.** Data extraction.

## Data Availability

All data generated or analysed during this study are included in this published article and its supplementary information files.
