# Supplementary figures and images for: Sex and gender considerations in reporting guidelines for health research: a systematic review
Source: Biol Sex Differ. 2021 Nov 20;12:62. doi: 10.1186/s13293-021-00404-0 (PMC8605583; doi:10.1186/s13293-021-00404-0)

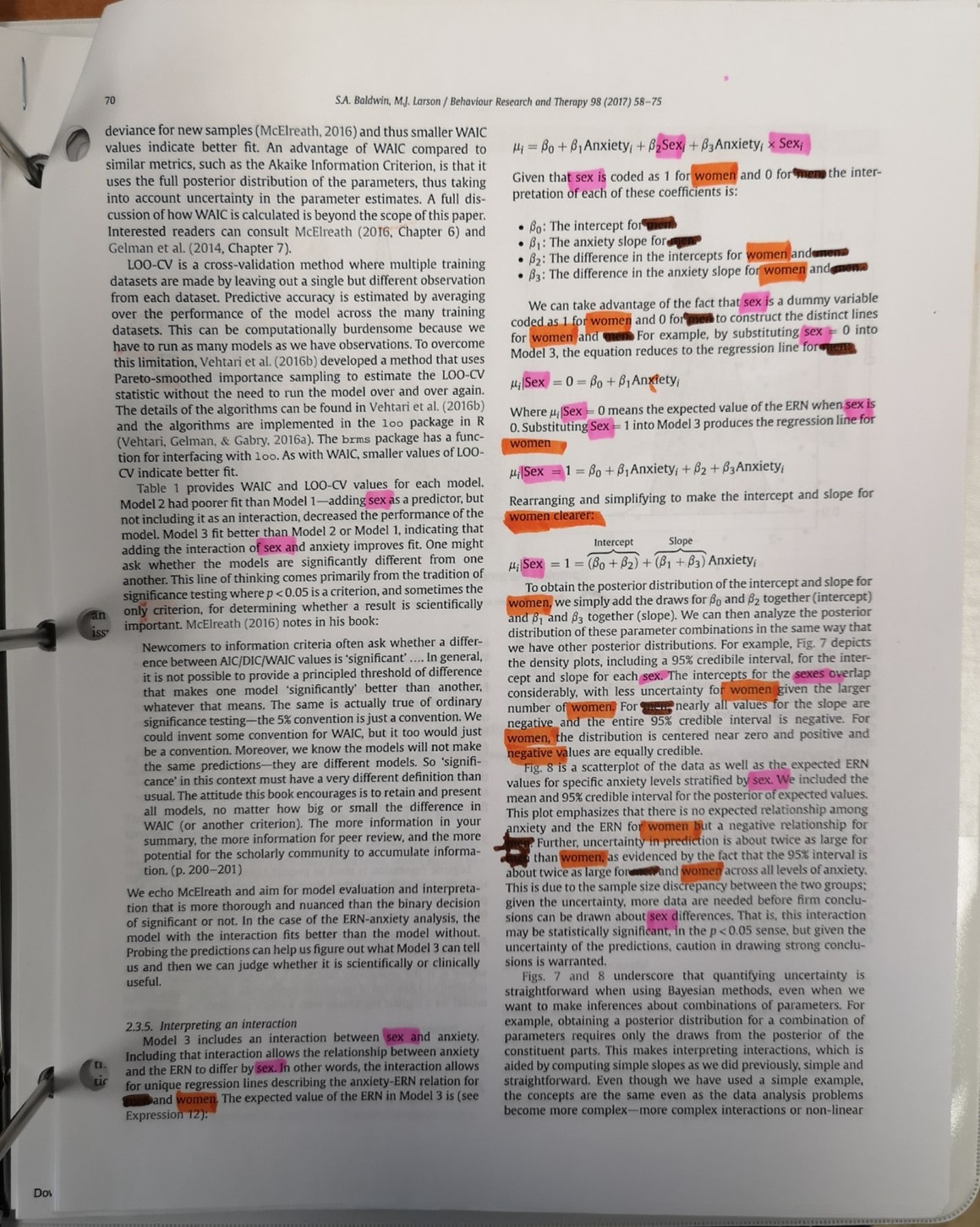

Supplement: Supplementary file 2 — Additional file 2: Fig. S1. Example of manual count of sex- and gender-related words. [file 13293_2021_404_MOESM2_ESM.jpg]

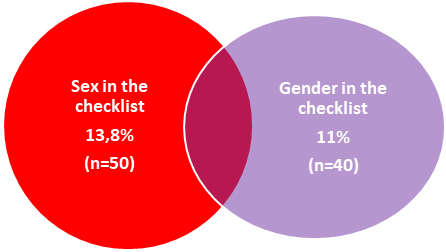


**1%**

**(n=4)**

**Guideline with checklist**

**n=363**

Figure S1. Use of sex and/or gender terms in the checklists

Supplement: Supplementary file 8 — Additional file 8: Fig. S1. Use of sex and/or gender terms in the checklists. [file 13293_2021_404_MOESM8_ESM.docx]
