# Supplementary material for: Sex and gender considerations in reporting guidelines for health research: a systematic review
Source: Biol Sex Differ. 2021 Nov 20;12:62. doi: 10.1186/s13293-021-00404-0 (PMC8605583; doi:10.1186/s13293-021-00404-0)
Supplement: Supplementary file 3 — Additional file 3. Validity assessment for methods systematic review. [file 13293_2021_404_MOESM3_ESM.docx]

**Internal validity assessment for methods systematic reviews^[[1]](#endnote-1)^**

1. Did the developers of the guideline represent more than one stakeholder group (e.g. researchers, funders, publishers)?

*Note: Look for anything about stakeholder consultations in the text of the paper.*

*An answer of yes = they explicitly reported more than one stakeholder group; if a specific group is named (e.g., the CONSORT Group), verify its composition on the internet.*

*An answer of no = they were explicit and reported that they did not represent other stakeholders. Otherwise answer = unclear.*

1. Did the developers report gathering any data for the creation of the guideline (e.g., carry out a literature review, collect anecdotal data)?

*Note: methods of data gathering must be clearly stated. Citations next to checklist items do not count as data gathering. Look for information in the text only.*

*An answer of yes = they were explicit mention of any type of data gathering*

*An answer of no = they were explicit and reported that they did not gather any data. Otherwise answer = unclear.*

1. Did the developers report the use of a consensus process (e.g., Delphi, RAND/UCLA Appropriateness Method, nominal group technique, consensus, meeting, development conference, roundtable, panel, in-person discussion)?

*Note:*

*An answer of yes = they were explicit and reported any of the consensus activities listed above*

*An answer of no = they were explicit and reported that they did not conduct any consensus activities. Otherwise answer = unclear.*

1. Adapted from Cukier S, Helal L, Rice DB, Pupkaite J, Ahmadzai N, Wilson M, et al. Checklists to detect potential predatory biomedical journals: a systematic review. BMC Med. 2020; 18(1):104.

   **Goal**: To assess evidence-based development of the reporting guideline

   **Scoring**: “yes” (i.e. low risk of bias), “no” (i.e. high risk of bias) or “unclear” (i.e. unclear risk of bias).

   **Overall**: high internal validity (low risk of bias): ≥ 2 “yes”

   low internal validity (high risk of bias): < 2 “yes” [↑](#endnote-ref-1)
