# Supplementary material for: Sex and gender considerations in reporting guidelines for health research: a systematic review
Source: Biol Sex Differ. 2021 Nov 20;12:62. doi: 10.1186/s13293-021-00404-0 (PMC8605583; doi:10.1186/s13293-021-00404-0)
Supplement: Supplementary file 5 — Additional file 5. Table S2. Comparison of electronic and manual identification of sex and gender related words in reporting guidelines. [file 13293_2021_404_MOESM5_ESM.docx]

S2 Table. Comparison of electronic and manual identification of sex and gender related words in reporting guidelines

| Id | Checklist | | | | | | Statement | | | | | | | | | | References | | | | | | | |
| --- | --- | --- | --- | --- | --- | --- | --- | --- | --- | --- | --- | --- | --- | --- | --- | --- | --- | --- | --- | --- | --- | --- | --- | --- |
|  | **Sex** | | **Men** | | **Women** | | **Gender** | | **Male** | | **Woman** | | **Men** | | **Women** | | **Sex** | | **Gender** | | **Men** | | **Women** | |
|  | **M** | **E** | **M** | **E** | **M** | **E** | **M** | **E** | **M** | **E** | **M** | **E** | **M** | **E** | **M** | **E** | **M** | **E** | **M** | **E** | **M** | **E** | **M** | **E** |
| 37 |  |  | 0 | **1** | 0 | **1** |  |  |  |  |  |  |  |  |  |  |  |  |  |  |  |  |  |  |
| 50 |  |  |  |  |  |  |  |  |  |  |  |  | 0 | **3** | 0 | **4** |  |  |  |  |  |  |  |  |
| 60 |  |  |  |  |  |  |  |  |  |  |  |  | 14 | **15** |  |  |  |  |  |  |  |  |  |  |
| 79 |  |  |  |  |  |  | 0 | **1** |  |  |  |  |  |  |  |  |  |  |  |  |  |  |  |  |
| 123 |  |  |  |  |  |  |  |  |  |  |  |  |  |  |  |  | 0 | **1** |  |  |  |  |  |  |
| 129 |  |  |  |  |  |  |  |  |  |  | 0 | 2 |  |  |  |  |  |  |  |  |  |  |  |  |
| 189 |  |  |  |  |  |  |  |  | 3 | **4** |  |  |  |  |  |  |  |  |  |  |  |  |  |  |
| 263 |  |  |  |  |  |  | 1 | **3** |  |  |  |  | 0 | **2** |  |  |  |  | **7** | 6 |  |  |  |  |
| 303 |  |  |  |  |  |  |  |  |  |  |  |  |  |  |  |  |  |  |  |  | 0 | **1** | 0 | **1** |
| 311 | 0 | **1** |  |  |  |  |  |  |  |  |  |  |  |  |  |  |  |  |  |  |  |  |  |  |
| 323 |  |  |  |  |  |  |  |  |  |  |  |  |  |  |  |  | 0 | **1** |  |  |  |  |  |  |
| 385 |  |  |  |  |  |  |  |  |  |  |  |  |  |  | 4 | **2** |  |  |  |  |  |  |  |  |

E: Electronic search; M : manual search; correct value in green after verification
