# Supplementary material for: Sex and gender considerations in reporting guidelines for health research: a systematic review
Source: Biol Sex Differ. 2021 Nov 20;12:62. doi: 10.1186/s13293-021-00404-0 (PMC8605583; doi:10.1186/s13293-021-00404-0)
Supplement: Supplementary file 6 — Additional file 6. Table S3. Concordance between electronic and manual identification of sex and gender related words in reporting guidelines. [file 13293_2021_404_MOESM6_ESM.docx]

S3 Table. Concordance between electronic and manual identification of sex and gender related words in reporting guidelines

|  | **Electronic** | | | | **Manual** | | | |  |
| --- | --- | --- | --- | --- | --- | --- | --- | --- | --- |
| **Characteristics** | | | | | | | | | |
|  | Min_e_ | Max_e_ | Mean_e_ | Med_e_ | Min_m_ | Max_m_ | Mean_m_ | Med_m_ | Conc % |
| **Checklist** | | | | | | | | | |
| Sex | 0 | 1 | 0.02439 | 0 | 0 | 0 | 0 | 0 | 97.6 |
| Gender | 0 | 3 | 0.12195 | 0 | 0 | 3 | 0.12195 | 0 | 100 |
| Woman | 0 | 0 | 0 | 0 | 0 | 0 | 0 | 0 | 100 |
| Women | 0 | 1.0000 | 0.02439 | 0 | 0 | 0 | 0 | 0 | 97.6 |
| Man | 0 | 0 | 0 | 0 | 0 | 0 | 0 | 0 | 100 |
| Men | 0 | 1.0000 | 0.02439 | 0 | 0 | 0 | 0 | 0 | 97.6 |
| Female | 0 | 1.0000 | 0.04878 | 0 | 0 | 1.0000 | 0.04878 | 0 | 100 |
| Girl | 0 | 0 | 0 | 0 | 0 | 0 | 0 | 0 | 100 |
| Boy | 0 | 0 | 0 | 0 | 0 | 0 | 0 | 0 | 100 |
| Male | 0 | 1.0000 | 0.04878 | 0 | 0 | 1.0000 | 0.04878 | 0 | 100 |
| Total |  | | | | | | | | 99.3 |
| **Abstract** | | | | | | | | | |
| Sex | 0 | 0 | 0 | 0 | 0 | 0 | 0 | 0 | 100 |
| Gender | 0 | 0 | 0 | 0 | 0 | 0 | 0 | 0 | 100 |
| Woman | 0 | 0 | 0 | 0 | 0 | 0 | 0 | 0 | 100 |
| Women | 0 | 0 | 0 | 0 | 0 | 0 | 0 | 0 | 100 |
| Man | 0 | 0 | 0 | 0 | 0 | 0 | 0 | 0 | 100 |
| Men | 0 | 0 | 0 | 0 | 0 | 0 | 0 | 0 | 100 |
| Female | 0 | 0 | 0 | 0 | 0 | 0 | 0 | 0 | 100 |
| Girl | 0 | 0 | 0 | 0 | 0 | 0 | 0 | 0 | 100 |
| Boy | 0 | 0 | 0 | 0 | 0 | 0 | 0 | 0 | 100 |
| Male | 0 | 0 | 0 | 0 | 0 | 0 | 0 | 0 | 100 |
| Total |  | | | | | | | | 100 |
| **Statement** | | | | | | | | | |
| Sex | 0 | 30 | 0.90243 | 0 | 0 | 30 | 0.90243 | 0 | 100 |
| Gender | 0 | 4 | 0.34146 | 0 | 0 | 4 | 0.26829 | 0 | 95.1 |
| Woman | 0 | 2 | 0.04878 | 0 | 0 | 0 | 0 | 0 | 97.6 |
| Women | 0 | 17 | 0.75609 | 0 | 0 | 17 | 0.70731 | 0 | 95.1 |
| Man | 0 | 0 | 0 | 0 | 0 | 0 | 0 | 0 | 100 |
| Men | 0 | 15 | 0.56097 | 0 | 0 | 14 | 0.41463 | 0 | 95.1 |
| Female | 0 | 2 | 0.07317 | 0 | 0 | 2 | 0.07317 | 0 | 100 |
| Girl | 0 | 0 | 0 | 0 | 0 | 0 | 0 | 0 | 100 |
| Boy | 0 | 0 | 0 | 0 | 0 | 0 | 0 | 0 | 100 |
| Male | 0 | 4 | 0.14634 | 0 | 0 | 3 | 0.12195 | 0 | 97.6 |
| Total |  | | | | | | | | 98.1 |
| **References** | | | | | | | | | |
| Sex | 0 | 1 | 0.12195 | 0 | 0 | 1 | 0.07317 | 0 | 95.1 |
| Gender | 0 | 6 | 0.14634 | 0 | 0 | 7 | 0.17073 | 0 | 97.6 |
| Woman | 0 | 0 | 0 | 0 | 0 | 0 | 0 | 0 | 100 |
| Women | 0 | 9 | 0.43902 | 0 | 0 | 9 | 0.41463 | 0 | 97.6 |
| Man | 0 | 0 | 0 | 0 | 0 | 0 | 0 | 0 | 100 |
| Men | 0 | 2 | 0.09756 | 0 | 0 | 2 | 0.07317 | 0 | 97.6 |
| Female | 0 | 0 | 0 | 0 | 0 | 0 | 0 | 0 | 100 |
| Girl | 0 | 0 | 0 | 0 | 0 | 0 | 0 | 0 | 100 |
| Boy | 0 | 1 | 0.02439 | 0 | 0 | 1 | 0.02439 | 0 | 100 |
| Male | 0 | 0 | 0 | 0 | 0 | 0 | 0 | 0 | 100 |
| Total |  |  |  |  |  |  |  |  | 98.8 |

Min : minimum ; Max : maximum ; Med : median ; Conc: concordance
