# Supplementary material for: Sex and gender considerations in reporting guidelines for health research: a systematic review
Source: Biol Sex Differ. 2021 Nov 20;12:62. doi: 10.1186/s13293-021-00404-0 (PMC8605583; doi:10.1186/s13293-021-00404-0)
Supplement: Supplementary file 9 — Additional file 9: Table S4. Distribution of the use of “sex” in various study types and sections of reporting guidelines. [file 13293_2021_404_MOESM9_ESM.docx]

Table S4. Distribution of the use of “sex” in various study types and sections of reporting guidelines

|  | **Section of reporting guideline** | | | |  |
| --- | --- | --- | --- | --- | --- |
|  | Checklist | Flowchart | Abstract | Statement | All^1^ |
| **Study type** | | | | |  |
| Case report^2^ | 1 (25) | 0 (0) | 0 (0) | 0 (0) | 1 (25) |
| Clinical practice guideline | 0 (0) | 0 (0) | 0 (0) | 0 (0) | 0 (0) |
| Diagnostic/prognostic | 8 (47.6) | 0 (0) | 0 (0) | 2 (11.1) | 9 (50) |
| Economic evaluation | 0 (0) | 0 (0) | 0 (0) | 2 (12.5) | 3 (18.8) |
| Experiment | 11 (8.6) | 0 (0) | 0 (0) | 18 (12.3) | 30 (29.4) |
| Nonspecific^3^ | 12 (14.5) | 1 (25) | 1 (1.3) | 15 (14.6) | 24 (23.3) |
| Observational | 21 (18.4) | 0 (0) | 1 (0.9) | 18 (4.4) | 39 (32.5) |
| Other^4^ | 0 (0) | 0 (0) | 0 (0) | 2 (11.8) | 3 (17.7) |
| Preclinical | 5 (1.4) | 0 (0) | 1 (7.7) | 4 (26.7) | 8 (53.3) |
| Protocol | 0 (0) | 0 (0) | 0 (0) | 1 (10) | 1 (10) |
| Quality improvement | 0 (0) | 0 (0) | 0 (0) | 0 (0) | 0 (0) |
| Qualitative | 1 (6.3) | 0 (0) | 0 (0) | 1 (6) | 3 (17.7) |
| Randomised trial | 10 (2.6) | 0 (0) | 0 (0) | 17 (12.8) | 29 (21.8) |
| Systematic review | 5 (1.4) | 0 (0) | 0 (0) | 5 (14.7) | 8 (23.5) |
|  |  |  |  |  |  |

^1^All sections including references

^2^Based on category of study types of EQUATOR homepage

^3^ Do not apply to any specific type of study

^4^As specified on the individual page of reporting guidelines on EQUATOR
