# Supplementary material for: Sex and gender considerations in reporting guidelines for health research: a systematic review
Source: Biol Sex Differ. 2021 Nov 20;12:62. doi: 10.1186/s13293-021-00404-0 (PMC8605583; doi:10.1186/s13293-021-00404-0)
Supplement: Supplementary file 10 — Additional file 10: Table S5. Distribution of the use of “gender” in various study types and sections of reporting guidelines. [file 13293_2021_404_MOESM10_ESM.docx]

Table S5. Distribution of the use of “gender” in various study types and sections of reporting guidelines

|  | **Section of reporting guideline** | | | |  |
| --- | --- | --- | --- | --- | --- |
|  | Checklist | Flowchart | Abstract | Statement | All^1^ |
| **Study type** | | | | |  |
| Case report^2^ | 3 (75.0) | 0 (0) | 0 (0) | 0 (0) | 3 (75.0) |
| Clinical practice guideline | 0 (0) | 0 (0) | 0 (0) | 2 (25) | 2 (25) |
| Diagnostic/prognostic | 1 (5.9) | 0 (0) | 0 (0) | 3 (16.7) | 4 (22.2) |
| Economic evaluation | 0 (0) | 0 (0) | 0 (0) | 2 (12.5) | 2 (12.5) |
| Experiment | 9 (7.0) | 0 (0) | 0 (0) | 21 (14.4) | 25 (17.1) |
| Nonspecific^3^ | 9 (2.5) | 1 (25) | 4 (5.2) | 16 (15.5) | 22 (21.4) |
| Observational | 14 (12.3) | 0 (0) | 0 (0) | 26 (21.2) | 31 (25.8) |
| Other^4^ | 2 (12.5) | 0 (0) | 0 (0) | 4 (23.5) | 6 (35.3) |
| Preclinical | 3 (23.1) | 0 (0) | 1 (7.7) | 3 (20) | 5 (33.3) |
| Protocol | 2 (20) | 0 (0) | 0 (0) | 2 (20) | 3 (30) |
| Quality improvement | 0 (0) | 0 (0) | 0 (0) | 1 (20) | 1 (20) |
| Qualitative | 2 (12.5) | 0 (0) | 0 (0) | 4 (23.5) | 4 (23.5) |
| Randomised trial | 7 (6.1) | 0 (0) | 0 (0) | 18 (13.5) | 22 (16.5) |
| Systematic review | 3 (8.8) | 0 (0) | 0 (0) | 7 (20.6) | 9 (26.5) |
|  |  |  |  |  |  |

^1^All sections including references

^2^Based on category of study types of EQUATOR homepage

^3^ Do not apply to any specific type of study

^4^As specified on the individual page of reporting guidelines on EQUATOR
