# Supplementary material for: Sex and gender considerations in reporting guidelines for health research: a systematic review
Source: Biol Sex Differ. 2021 Nov 20;12:62. doi: 10.1186/s13293-021-00404-0 (PMC8605583; doi:10.1186/s13293-021-00404-0)
Supplement: Supplementary file 11 — Additional file 11: Fig. S2. Publication trends in the mean number of occurrences of "sex" and "gender" in the checklist. [file 13293_2021_404_MOESM11_ESM.docx]

Figure S2. Publication trends in the mean number of occurrences of “sex” and “gender” in the checklist
